# Maryland

**Published:** 1897-03

**Authors:** 


*Maryland.* Secretary Clement writes, in reply to interrogatory on the subject: Our examining board have not taken up the question of dehorning cattle by non-registered persons for two reasons: first, on account of the peculiar reading of our laws relative to the State outside of the city of Baltimore; second, because we consider it hardly a surgical operation, but a proceeding which can just as well be accomplished by one man as another, provided he possesses the proper apparatus.

